# Biomarker-based assessment of cerebral small vessel disease progression after ischemic stroke – a prospective cohort study (MA-BIO)

**DOI:** 10.1186/s42466-026-00514-3

**Published:** 2026-08-17

**Authors:** Lachin Hasanova, Paula Klassen, Anastasia Nosanova, Malak Babayeva, Dorothée Lulé, Hayrettin Tumani, Hans-Peter Müller, Deniz Yilmazer-Hanke, Nico Sollmann, Kornelia Kreiser, Karl Georg Haeusler, Mona Laible

**Affiliations:** 1 Neurology Department, Vascular Division, Neurology Clinic, University Hospital Ulm, Ulm, Germany; 2 Institute for Anatomy and Cell Biology, Ulm University, Ulm, Germany; 3 Department of Nuclear Medicine, University Hospital Ulm, Ulm, Germany; 4 Department of Diagnostic and Interventional Radiology, University Hospital Ulm, Ulm, Germany; 5 Clinical Neuroanatomy, Neurology Department, Vascular Division, University Hospital Ulm, Ulm, Germany; 6 Department of Diagnostic and Interventional Neuroradiology, School of Medicine and Health, TUM Klinikum Rechts der Isar, Technical University of Munich, Munich, Germany; 7 TUM-Neuroimaging Center, TUM Klinikum Rechts der Isar, Technical University of Munich, Munich, Germany; 8 Department of Neurology, School of Medicine, Ulm University, Campus Nord, Oberer Eselsberg 45, Ulm, 89081 Germany

**Keywords:** Stroke, Cerebral small vessel disease, Biomarkers, Magnetic resonance imaging, Cognitive assessment

## Abstract

**Background:**

Cerebral small vessel disease (CSVD) is a leading cause of ischemic stroke and vascular cognitive impairment, yet currently lacks clinically established biomarkers for monitoring disease activity. While clinical routine magnetic resonance imaging (MRI) can provide quantitative measures of structural brain changes, it does not capture ongoing pathophysiological processes. Blood-based biomarkers offer a minimally invasive approach to detect dynamic changes in CSVD and may provide novel insights into mechanisms of progression and potential targets for intervention. This study aims to evaluate longitudinal trajectories of candidate blood biomarkers and their association with MRI-derived markers and cognitive outcomes in patients with CSVD.

**Methods:**

In this single-center, prospective observational cohort study at the University Hospital Ulm, Germany, up to 180 adults with MRI-proven evidence of CSVD will be recruited and stratified into three groups: (1) CSVD without acute ischemic stroke, (2) CSVD with acute lacunar ischemic stroke, and (3) CSVD with territorial ischemic stroke. Participants will undergo comprehensive assessments at baseline, at 3, 6, and 12 months after enrollment. The primary outcome is defined as longitudinal changes of White Matter Hyperintensities (WMH) volumes. Secondary outcomes upon one year include DTI-derived measures, changes in blood biomarkers, cognitive performance, clinical functional measures, and incidence of new cerebrovascular events. Imaging, neurovascular ultrasound, and neuropsychological assessments will be conducted using standardized protocols. Clinical primary and secondary outcomes will be correlated to post-mortem CSVD pathology in the brain in prospectively studied patients, who have consented to autopsy.

**Discussion:**

By integrating blood-based biomarkers with advanced imaging and longitudinal cognitive assessments, this study aims to advance our understanding of CSVD pathophysiology and may identify biomarkers of disease activity. Our findings hopefully inform future precision-medicine approaches and novel therapeutic strategies.

**Trial registration:**

German Clinical Trials Registry (DRKS00038936), registered February 3, 2026.

## Introduction

Cerebral small vessel disease (CSVD) is a major cause of ischemic stroke and contributes substantially to vascular cognitive impairment and subsequent dementia, accounting for approximately one quarter of ischemic strokes and vascular dementias [1]. Key neuroimaging correlates of CSVD include white matter hyperintensities (WMH), cerebral microbleeds, lacunes, perivascular spaces, and superficial siderosis, which can be assessed by brain magnetic resonance imaging (MRI) [1, 2]. Hence, brain MRI is considered the diagnostic reference standard and also allows quantitative assessment of CSVD burden, including volumetric evaluations [2, 3]. Advanced MRI techniques such as diffusion tensor imaging (DTI), amongst other techniques, can enable further characterization of microstructural white matter injury beyond conventional WMH assessment [4, 5].

Recent neuroimaging studies indicated that CSVD may show mild partial regression in an observation over several years, highlighting the dynamic nature of the disease [6, 7]. From a therapeutic perspective, evidence supports antihypertensive treatment as a secondary preventive strategy [1, 4]. In addition, randomized clinical trials suggest that pharmacological approaches such as the combination of cilostazol and isosorbide mononitrate may reduce stroke recurrence and improve functional and cognitive outcomes, although the generalizability of these findings is uncertain [8]. Longitudinal observational studies suggest that WMH progression may be accelerated following lacunar stroke compared with asymptomatic individuals, particularly in patients with higher baseline CSVD burden [9, 10]. With higher degrees of dysconnectivity as a result of acute stroke and preexisting CSVD affecting deep WM, functional outcome is worse [11]. However, available data are based on datasets of observational and interventional prospective trials on CSVD or ischemic stroke and do not adequately clarify how stroke mechanism, size, and localization impact on CSVD progression [12, 13].

Beyond brain MRI, no clinically established in vivo surrogate marker for CSVD activity currently exists. Blood-based biomarkers represent a promising approach for monitoring longitudinal disease activity and potential regression. Of those, serum neurofilament light chain (NfL) levels seem in particular promising as they have been found to reflect imaging burden and neuropsychological features (processing speed performance) of CSVD in a cross-sectional study including more than 500 patients [14]. Furthermore, temporal changes of serum NfL have been described in a monocenter cohort of 79 patients after a recent small subcortical infarct upon 15 months. Compared to healthy controls, NfL levels were increased shortly after stroke and until 3 months, returning to normal levels after 15 months [15]. New incident ischemic lesions on MRI were reflected by secondary NfL increases [15]. Further results from the Alzheimer’s Disease Neuroimaging Initiative (ADNI) showed that plasma NfL correlated positively with CSVD severity, and with the presence of cerebral microbleeds (CMBs) and lacunes [16]. With higher the changes in plasma-NfL were over time, the CSVD burden and WMH increased upon a mean follow-up of 27 months in 378 patients [16]. These findings imply an additional value of blood NfL measurements for disease monitoring, which may be complementary to brain MRI.

In a monocenter, prospective registry, serum GFAP correlated with verbal fluency, visuomotor and executive functions in patients with CSVD [17]. Lately, in a follow-up study over 12 months, vascular endothelial growth factor (VEGF) levels in the blood were associated with incident brain lesions in a longitudinal study [18]. Beyond that, such biomarkers have demonstrated substantial diagnostic and longitudinal value in other neurological disorders, including multiple sclerosis and neurodegenerative diseases [19, 20].

The present prospective cohort study builds upon a previously conducted single-center pilot study by our group (local Ethics approval no. 343/22), which demonstrated the feasibility of longitudinal biomarker measurements in patients with CSVD and provided preliminary evidence supporting associations between biomarker dynamics and MRI-detected CSVD progression.

The objectives of the presented study include the identification of one blood biomarker or a set of biomarkers that can help identify disease progression in CSVD. A further aim is to describe differences of biomarker courses in direct comparison of patients with CSVD and acute lacunar ischemia with patients with territorial ischemia in a prospective, consecutive and multi-modal trial.

### Primary outcome

The primary outcome is the change in WMH volume based on brain MRI measurements.

### Secondary outcomes

Secondary outcomes include changes of DTI-derived measures, blood biomarker quantifications, in neuropsychological test results over time, novel cerebral ischemic events, myocardial infarction, the clinical disease course as marked by the modified Rankin Scale score (mRS) and quality of life upon 12 months.

### Post-mortem study outcomes

As part of a translational approach, study participants will be asked for a post-mortem donation of their brain for scientific purposes. Outcomes of such postmortem studies include (i) routine neuropathological diagnostics including staging of neurodegenerative changes, (ii) microangiopathy classification and scoring including arterio- and arteriolosclerosis, blood-brain-barrier disturbances, glio-neuronal changes, WMH-related WM pathology, string vessels, vascular bagging, and vascular beta-amyloid (Aβ) accumulation, as well as (iii) size, type (ischemic / hemorrhagic), and location of territorial infarcts, microinfarcts, microbleeds, lacunes, and superficial siderosis.

## Methods

### Participants

#### Study design

The study is a registered (DRKS00038936; date of registration 01.02.2026) investigator-initiated single-center, prospective, observational study with an expected total study duration of 3 years.

The study is conducted at the University Hospital of Ulm, Ulm, Germany. As a tertiary care hospital in southern-central Germany, it serves large parts of southeastern Baden-Württemberg, as well as adjacent regions in Western Bavaria. Participants are recruited at the stroke unit, the normal wards of the Department of Neurology, and neurological outpatients clinics. Patient recruitment started in March 2026. Prior, we conducted a pilot trial, for which recruitment begun in March 2023 with the last patient visit of 29 completely followed-up patients in May 2026 (unpublished data).

At the Department of Neurology, University Hospital Ulm, we treat about 1000 patients with ischemic stroke/transient ischemic attack per year. In approximately one fifth of such cases (*n* = 200), we anticipate a microangiopathic stroke etiology, as confirmed by MRI findings. A territorial stroke etiology is expected with a similar frequency. Furthermore, the neurological clinic annually manages several hundred patients with CSVD, but without evidence of recent cerebral ischemia.

### Eligibility criteria

Patients are eligible as participants if (a) their age is ≥ 18 years and if (b) brain MRI provides evidence of CSVD, defined as WMH (Fazekas grade 1–3). Patients with Fazekas grade 1 will be included as well to cover all different stages of CSVD and because even a mild lesion load has been linked to neurocognitive impairment [21]. In case of uncertainty about the aetiology of WMH, diagnostics to rule out inflammatory diseases or genetic small vessel diseases are planned. Furthermore, (c) patients must be assigned to one of the following groups based on MRI findings: Group 1: CSVD without evidence of an acute ischemic stroke, Group 2: CSVD with acute lacunar ischemic stroke, defined as singular diffusion-weighted imaging (DWI)-hyperintense, apparent diffusion coefficient (ADC)-hypointense lesion on brain MRI with a diameter of maximum of 2.5 cm, or group 3: CSVD with acute territorial ischemic stroke pattern on MRI. All patients must be (d) able to provide written informed consent and (e) have sufficient German language skills to understand study procedures and assessments. The requirement for sufficient German language skills is necessary to ensure valid neuropsychological assessments. However, this criterion may introduce selection bias and may limit the generalizability of the study findings.

Inclusion criteria are as follows:

- Age ≥ 18 years.
- Evidence of CSVD on brain MRI, defined as the presence of WMH (Fazekas grade 1–3).
- Assignment to one of the defined study groups based on MRI findings.
- Ability to provide written informed consent.
- Sufficient German language skills.

Exclusion criteria are as follows:

- Alternative plausible causes of WMH other than CSVD.
- Known neurodegenerative diseases.
- Acute or recent traumatic brain injury.
- Contraindications to MRI.
- Pregnancy or breastfeeding.
- Expected life expectancy of less than one year.
- Inability to comply with study procedures or follow-up visits.
- Severe renal insufficiency with an estimated glomerular filtration rate <15 ml/min

### Informed consent

All participants need to give their written informed consent to participate. The study is conducted according to the Declaration of Helsinki, its original version and its latter amendments with the latest amendment of 2024.

#### Sample size

We aim to include 60 patients in the three study groups, resulting in a maximum total number of 180 patients. We expect a drop-out proportion of 25%, relating to difficulties of mobility and morbidity for adherence in follow-up visits of patients with territorial brain ischemia in particular and considering the rural area surrounding the region of Ulm, Baden-Württemberg, Germany. The calculation of sample size is not based on a confirmatory study aim, but is exploratory and mirrors our experiences made in the pilot study of the recruitment that can be expected and delivered in our clinical setting in Ulm.

As an illustration of sensitivity: in a pilot subset (unpublished data) with paired manual WMH volumetry (17 patients), the mean 12-month WMH volume change was 5.30 mL (SD 6.03; standardized within-subject effect d_z ≈ 0.88). A within-group comparison detects a change of this magnitude at ~ 80% power (two-sided α = 0.05) in ~ 10 patients per group. With 60 patients per group (≈ 45 completing follow-up after the anticipated 25% drop-out), the planned cohort is sensitive to within-subject WMH changes as small as d_z ≈ 0.36 (at 60 per group) to 0.42 (at 45 per group), i.e. ≈ 2.2–2.5 mL, well below the 5.30 mL observed in the pilot data, and, for exploratory between-group comparisons, to standardized differences of d ≈ 0.51 (at 60 per group) to 0.59 (at 45 per group), a ≈ 3.1–3.6 mL difference in WMH change between groups.

### Recruitment

The recruitment is carried out by a designated study team of the Department of Neurology, University Hospital Ulm, Germany, during the in-patient stay of patients eligible for participation in Groups 2 and 3. Patients with CSVD without a current stroke are recruited at our specialized outpatient clinic for CSVD and cerebrovascular diseases. As of June 11th, 2026 6 male patients and 3 female patient were enrolled. Of those, 5 (56%) presented with acute territorial stroke and were recruited in Group 3, 3 had a lacunar stroke and were assigned to group 2 (33%) and one further participant without acute stroke was recruited into group 1 (11%). The median age was 68 years with a minimum of 52 and a maximum of 85 years.

### Demographics

For study participants, the following demographics and patient characteristics is collected: year of birth, sex (male/female/non-binary), ethnicity, International Standard Classification of Education (ISCED [22]), level, earliest/current profession/retirement according to International Standard Classification of Occupations (ISCO) classification [23], family history of stroke, myocardial infarction, peripheral artery disease, venous thromboembolism, CSVD, known or obtained genetic disorders or test results respectively in the study participant or first-degree relatives (coded as negative/positive/not informative).

Furthermore, we gather information about the current living situation (living independently in a single/couple/family household/in a nursing home), and the marital and parental status. We ask for concurrent malignancy (present/absent) and, if present, specify the type and staging results. In addition, the history and current presence of cardiovascular risk factors is taken (arterial hypertension, diabetes, current/past smoker/hypercholesterolemia/family history as stated above).

### Parameters

In participants with known CSVD, age at disease onset and past brain MRI results will be collected. For each patient, at each visit, the following clinical parameters will be recorded: (1) current global or focal neurological symptoms (e.g. subjectively evident memory disorders, gait disorders, dysarthria, focal motor or sensory symptoms, bladder dysfunction, dizziness/vertigo), those symptoms are summed up using the National Institutes of Health Stroke Scale (NIHSS) is a standardized 15-item neurological examination tool used by healthcare professionals to quantify acute stroke severity and monitor neurological changes and the modified Rankin Scale (mRS), a 6-point scale for measuring the degree of disability or dependence in the daily activities (2) current blood pressure, heart rate, peripheral Sp02 saturation, (3) height and body weight to calculate the body mass index.

### Magnetic resonance imaging

Brain imaging is conducted at the University Hospital, Ulm, Department for Radiology, Section of Neuroradiology at a 1.5-T or 3-T MRI system (MAGNETOM Vida, Siemens Healthineers, Erlangen, Germany). Imaging includes the following sequences: DWI, 3D fluid attenuated inversion recovery (3D-FLAIR), susceptibility-weighted imaging (SWI) and/or T2* imaging, 3D-T2-weighted imaging, T1-weighted Magnetization-Prepared Rapid Gradient Echo (MPRAGE), and time-of-flight MR angiography (TOF-MRA).

On the 1.5-T system (MAGNETOM Altea, Siemens Healthineers, Erlangen, Germany) the 3D-FLAIR is acquired sagittally with a voxel size of ~ 1.0 × 1.0 × 1.4 mm^3^ (reconstruction voxel: ~0.5 × 0.5 × 1.0 mm^3^, 160 slices; TR = 7000 ms, TE = 433 ms; flip angle = 120°).

If possible (e.g., compliant patient, no emergency setting), advanced sequences will be added to the protocol, such as arterial spin labeling (ASL), resting-state functional MRI (rs-fMRI), or mapping sequences. On the 1.5-T system (MAGNETOM Altea, Siemens Healthineers, Erlangen, Germany) the 3D-FLAIR is acquired sagittally at 0.49 × 0.49 × 1.0 mm (acquired matrix 512 × 512, 160 slices; TR/TE 7000/433 ms; flip angle 120°). The 1.0 mm slice thickness meets, and the acquired 0.49 mm in-plane resolution exceeds the ~ 1 mm isotropic resolution referenced by STRIVE-2 for structural imaging.

For visual detection of ischemic lesions, a standard diffusion-weighted sequence will be used with whole-brain coverage. In addition, on the 3-T system (MAGNETOM Vida, Siemens Healthineers, Erlangen, Germany), a dedicated additional DWI sequence for advanced diffusion analyses (e.g., fractional anisotrophy (FA), the peak width of skeletonized mean diffusivity (PSMD)) will be added according to the following parameters: echo-planar imaging (EPI) with simultaneous multi-slice (SMS) acceleration using a factor = 2, no phase Partial Fourier, acquisition in transversal orientation, slice thickness = 3 mm, acquisition / reconstruction voxel size = ~ 2 × 2 × 3 mm^3^, repetition time (TR) = 3100 ms, echo time (TE) = 104 ms, fat saturation (strong), 65 diffusion directions, three diffusion weightings (b = 0 s/mm², b = 1000 s/mm², and b = 2000 s/mm²), and phase encoding direction = A > P, with an acquisition duration < 10 min. In addition, a short analogous DWI sequence with phase encoding direction = P > A will be added (to be able to correct for geometric distortions), with an acquisition duration < 1 min.

Features of CSVD are determined according to STandards for ReportIng Vascular changes on nEuroimaging (STRIVE) criteria [2]. This will be conducted by trained (neuro-)radiologists and vascular neurologists considering the above-mentioned sequences (especially FLAIR, T1-weighted MPRAGE, DWI, and SWI or T2*). Interrater reliability will be calculated for the determined volume of CSVD, the presence or absence of cerebral microbleeds, lacunes, and perivascular spaces using a set of randomly selected patients.

### Postprocessing

FLAIR and the other structural sequences are acquired as 3D volumes and reformatted into orthogonal planes for review and further image processing. In a first step, T2 FLAIR, and DWI sequences are visually assessed using a PACS (Picture Archiving and Communication System; GE Healthcare, Chicago, Illinois, USA) to ensure adequate image quality. The images are then exported and stored as Digital Imaging and Communications in Medicine (DICOM) files for further processing. MRI data are processed and analyzed using two established analysis pipelines, the TIFT software, which enables MRI datasets composed of voxels to be reconstructed into a three-dimensional model [5], as well as a pipeline for analysis of the PSMD, a well-established and validated approach specifically developed for CSVD that explicitly accounts for partial volume contamination [24]. The brains are subsequently aligned along the AC–PC line (an axis extending from the anterior commissure to the posterior commissure) to correct for potential head tilting during image acquisition.

Voxel intensities are displayed using different thresholds to distinguish between WMH, microbleeds, the index stroke lesion, and normal brain tissue. This approach enables the selective identification of structures whose signal intensities fall within predefined threshold ranges, such as the DWI-hyperintense, ADC-hypointense core of the acute ischemic stroke or WMH.

**Fig. 1:**
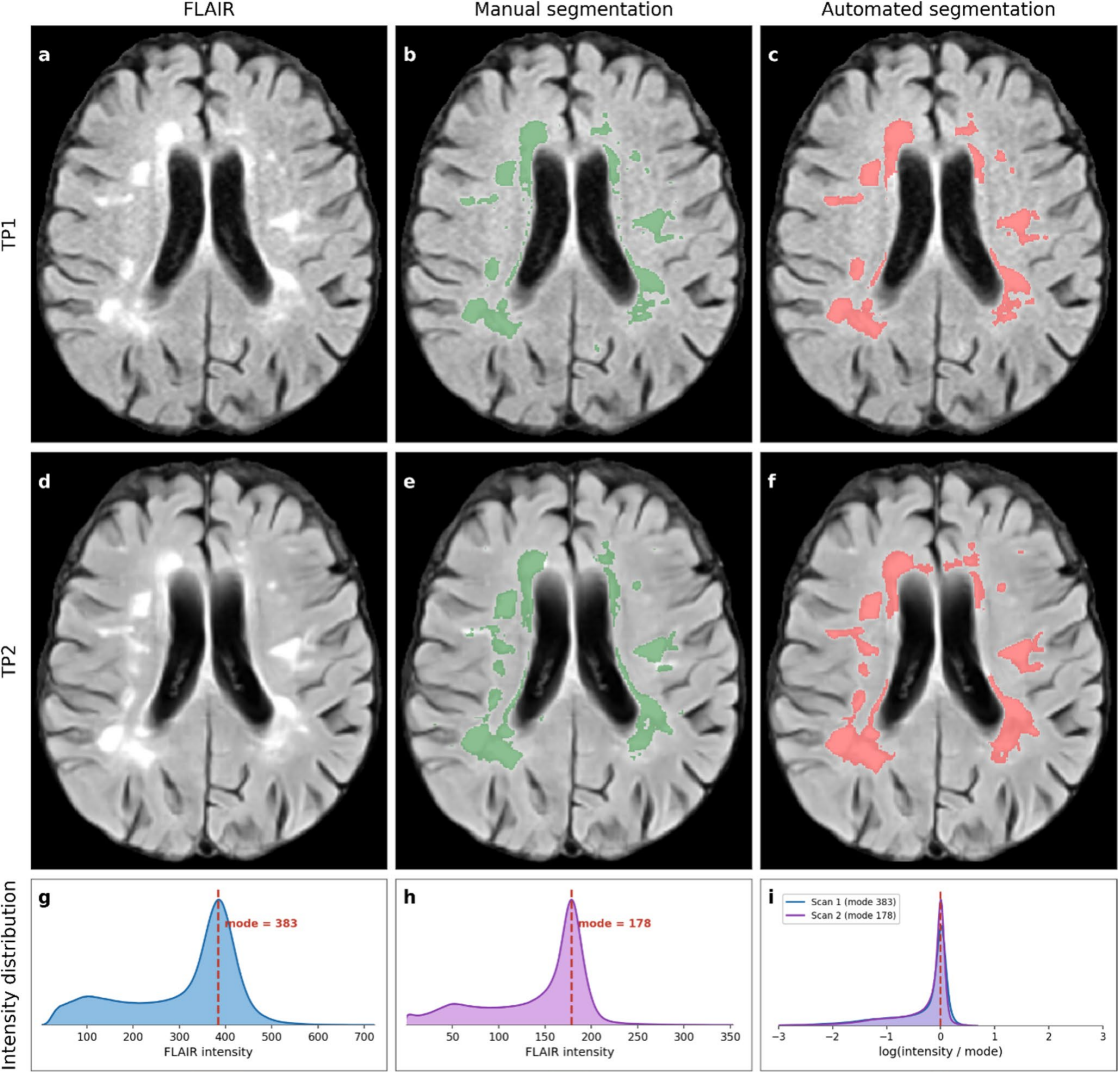
Examples of longitudinal white matter hyperintensity (WMH) segmentation across scanner sessions, as performed in participants of the pilot trial. (**a**, **d**) Timepoint 1 (TP1, baseline brain MRI) and timepoint 2 (TP2, 12 months follow-up brain MRI) fluid-attenuated inversion recovery (FLAIR) images. (**b**, **e**) Manual WMH segmentation (green overlay) for TP1 and TP2, respectively. (**c**, **f**) Automated segmentation using a hyperintense Gaussian mixture model (HGMM) pipeline (red overlay) for TP1 and TP2, respectively. (g) TP1 white-matter intensity distribution (mode = 383). (**h**) TP2 white-matter intensity distribution (mode = 178). (**i**) Log-normalized intensity distributions of both timepoints, confirming alignment of the mode-matched peaks across scanner sessions

Segmentation and quantification are then performed in three-dimensional space, allowing volumetric measurements as illustrated by examples in Fig. 1. DWI lesion volume and WMH volume are measured in mm³. All measurements will be performed by trained raters (KK, NS) following standardized training procedures. Raters performing volumetric assessments will be blinded to clinical information, neuropsychological results, and biomarker measurements.

To account for scanner differences between acquisitions, WMH segmentation will be performed using a FLAIR-only automated in-house pipeline based on the half-Gaussian mixture model described by Igwe et al. [25], which models log-transformed above-mode FLAIR intensities as a two-component mixture: a half-Gaussian for normal-appearing white matter and a full Gaussian for WMH, with all five parameters estimated via expectation-maximization. The original method was validated on a single scanner and timepoint; we extend it to longitudinal cross-scanner comparison by fitting the HGMM independently per timepoint on a shared white matter territory (intersection of both timepoints’ white matter labels), with a two-pass refinement that excludes candidate WMH from the fit pool to tighten the normal-appearing white matter model. Longitudinal change is estimated through paired voxel-wise contrast at the TP1 lesion boundary, corrected for scanner offset (estimated from stable white matter) and blur mismatch, rather than by subtracting two independent segmentations. Anatomical false positives are removed through automated exclusion. Acute DWI lesions identified on the baseline MRI were registered to FLAIR space andexcluded from the WMH volume calculations at baseline, 6-month, and 12-month follow-up. This procedure ensured that longitudinal WMH measurements represented chronic white matter disease burden, independent of the baseline acute infarct.

In the pilot cohort, baseline and follow-up FLAIR were acquired on different scanners and protocols, so standard endpoint WMH segmentation could not reliably separate true lesion change from scanner-induced contrast shifts. We therefore used a scanner-aware longitudinal pipeline designed to identify the FLAIR change as biological progression rather than scanner change artifacts. Since the prospective cohort is acquired on a stable, harmonized protocol, Deep WMH and Multi-site Auto-segmentation and Registration of Subcortical White Matter Hyperintensities (MARS)-WMH-derived segmentation will additionally be evaluated, with agreement to the manual reference quantified by Dice similarity coefficient and ICC.

### Neurovascular diagnostics

Furthermore, neurovascular duplex sonography of the extracranial and intracranial cerebral arteries is conducted at study visits, including assessment of intima–media thickness and arterial impedance. For interpretation of atherosclerotic plaques in the carotid arteries, the Mannheim Carotid Intima-Media Thickness and Plaque Consensus will be applied [26].

### Biosamples

At all scheduled study visits, venous blood samples will be collected, processed, aliquoted, and stored in the biobank of the Department of Neurology according to established standard operating procedures. Laboratory analyses include a comprehensive panel of vascular, metabolic, inflammatory, and neurodegeneration-related parameters. Vascular and metabolic markers comprise lipid parameters (Low- and high-Density Lipoprotein (LDL, HDL), cholesterol, triglycerides, and lipoprotein(a)) as well as markers of glucose metabolism, including insulin and glycated hemoglobin (HbA1c). Cardiac biomarkers (troponin I or T) at baseline in groups 2 and 3 and inflammatory markers, including high-sensitivity C-reactive protein, interleukin-6, and interleukin-1 alpha, are assessed to characterize systemic vascular and inflammatory status [27].In addition, blood-based biomarkers reflecting central nervous system injury are analyzed, e.g. GFAP as a marker of glial activation, neurofilament light chain (NfL) as a marker of axonal damage, phosphorylated tau 217 (pTau217) as an exploratory neurodegeneration-related biomarker, and beta-synuclein as an exploratory synaptic marker.

CSF samples obtained as part of routine clinical care and based on clinical indication (e.g. to exclude inflammation or vasculitis) will be used for biomarker analyses, including total tau protein, phosphorylated tau, and amyloid-β1–42. In addition, endothelial cell–related biomarkers, including endothelin-1 and vascular cell adhesion molecule-1 (VCAM-1), are analyzed to capture endothelial dysfunction associated with cerebral small vessel disease [28, 29].

### Cognitive and functional diagnostics and outcomes

1. Neuropsychological examinations are included to assess cognitive function longitudinally. We perform a neuropsychological assessment at the baseline visit and at the 12 months follow-up, which is adapted to the current capacity of the study participant with a focus on attention, executive function, visual and verbal primary and working memory, as well as visuospatial function. Furthermore, MoCA will be performed at each study visit. We elaborated two different scenarios of neuropsychological assessments according to the capacity of the respective participants as the acute stroke pattern, clinical impairment (e.g. fatigue, resilience, dysarthria, blood-pressure variations, impairment due to acute stroke treatment) and pre-stroke morbidity can differ substantially. In patients with expected ability to undergo extended neuropsychology, the following tests are performed: TAP to assess alertness; the executive basic function/visual fluency using the 5-Point Test according to Regard and verbal fluency. We use the Rey Complex Figure to assess for visuospatial function and visual memory; for verbal memory, RBANS is applied and for verbal intelligence we perform a vocabulary test.
2. In patients with low expected capacity for neuropsychological assessment, only the MoCA is carried out. Furthermore, MoCA is applied at each visit in its versions A-C.

The cognitive and functional outcome assessment is summarized in Table 1.

**Table 1:** Cognitive assessment as per study protocol

Neuropsychological assessment				
Visits	1	2	3	4
	Screening and Baseline	Follow-up	Follow-up	End-of-Study
Months after enrollment	0	3	6	12
Assessment				
Basic NPA	x			X
MoCA	x	x	x	X
Extended NPA	x			X
TAP Alertness	(x)			(x)
5-Point Test according to Regard	(x)			(x)
Rey Complex Figure	(x)			(x)
RBANS	(x)			(x)
Verbal fluency Test	(x)			(x)

MoCA, Montreal Cognitive Assessment; NPA, Neuropsychological Assessment; RBANS, Repeatable Battery for the Assessment of Neuropsychological Status; TAP, Test of Attentional Performance; Assessments in parenthesis display optional study elements

### Functional neurological assessment

Participants undergo clinical assessment using the NIHSS and mRS. Furthermore, we will gather information about their quality of life (QoL), using the structured EuroQol 5 Dimension (EQ-5D) [29]. The functional abilities of daily life are further specified using the activities of daily living (ADL) and the instrumental activities of daily living (IADL). In addition, and to capture influences of CSVD and stroke on the preservation of abilities to work, data will be collected using the Work Ability Index (WAI).

### Plans to promote participant retention and complete follow-up

In order to enhance participant retention, follow-up visits are coordinated to reduce burden and optimize adherence. The scheduled MRIs are performed in conjunction with study visits, accordingly, we can avoid additional appointments for the sole purpose of MRI. MRI is performed on a scanner next to the outpatient department. Participants receive direct DECT telephone contact numbers and email addresses of the study team members.

A complete and condensed overview of the study visits and procedures is given in Table 2.

**Table 2:** Study assessments alongside the visits 1-4

Schedule of assessments				
Visits	1	2	3	4
	Screening and baseline	Follow-up	Follow-up	End-of-study
Months after enrollment	0	3	6	12
Assessment				
Informed Consent	x			
Medical History	x	x	x	x
NIHSS	x	x	x	x
mRS	x	x	x	x
MRI	x		x	x
Basic NPA	x	x	x	x
Extended NPA	x			x
NVDU	x	x	x	x
Biomarkers	x	x	x	x
Blood Laboratory	x	x	x	x

NIHSS, National Institutes of Health Stroke Scale, mRS, modified Rankin Scale; MRI, Magnetic Resonance Imaging; EQ-5D, EuroQol 5 Dimension; extended NPA, Neuropsychologic Assessment; NVDU, neurovascular Doppler ultrasound; biomarkers: refers to blood-based biomarkers

### Benefit–risk assessment

Participation in the study is associated with low additional risks. The potential risks are minimal and mainly related to blood sampling and MRI examinations [2]. The MRI examinations are conducted without contrast agents, and all participants are comprehensively informed about potential discomforts and contraindications during the consent process.

There is no direct therapeutic benefit for participants. However, participants and/or their treating physicians may receive additional diagnostic information regarding disease progression and corresponding clinical counseling.

Given the minimal procedural risks and the substantial scientific value of identifying blood-based biomarkers for CSVD activity and progression, the overall benefit–risk ratio of the study is considered favorable.

### Data collection and management

Data will be collected and managed using REDCap (Research Electronic Data Capture), a secure, web-based electronic data capture platform. REDCap provides role-based access control, audit trails, automated data validation rules, and secure data storage. Study data will be entered directly into electronic case report forms (eCRFs) by authorized study personnel. Regular data quality checks and plausibility controls will be performed throughout the study to ensure data completeness, accuracy, and traceability in accordance with Good Clinical Practice (GCP) principles.

### Data management and confidentiality

Participants data will be pseudonymized and stored at a local in-house password-protected server. The server protection strategy is provided according to critical infrastructures (KRITIS) standards (https://www.bsi.bund.de/DE/Themen/Regulierte-Wirtschaft/Kritische-Infrastrukturen/Allgemeine-Infos-zu-KRITIS/allgemeine-infos-zu-kritis_node.html). Access to personal study participant data is provided to members of the study team in exclusion. Radiologic assessment is performed after data pseudonymization. In case that a participant withdraws from study participation, he/she will have the option to have all study-related data deleted. All study documents are stored securely for up to ten years after the closure of the registry. Data protection follows the German Federal Data Protection Act. A contact to the federal data protection authority is provided to all participants (www.baden-wuerttemberg.datenschutz.de/kontakt-aufnehmen/*).* Participants can review their data, request copies or corrections and communicate complaints or concerns with authorities.

### Statistical methods

#### Methods of analyses

The statistical analysis will be purely descriptive. Categorical variables will be summarized using absolute and relative frequencies, while continuous variables will be described using the mean and standard deviation or the median, interquartile range, and minimum and maximum values, as appropriate. Group differences will be assessed according to the measurement scale and distribution using the t-test or Wilcoxon signed rank test for continuous variables, and the chi-square test or Fisher’s exact test for categorical variables. To investigate temporal changes in parameters, individual slopes will first be estimated using linear mixed-effects models with random intercepts for participants to account for within-subject correlation. They will be subsequently compared between groups. In a further step, notable group differences will be explored using appropriate regression methods (linear regression, logistic regression, and, if applicable, mixed-effects models).

Differences in follow-up imaging with regard to the number and volume of radiological markers of CSVD will be analyzed using the Wilcoxon rank-sum test. Associations between continuous variables will be examined using scatter plots; where appropriate, Pearson or Spearman correlation coefficients will be calculated. Longitudinal data (e.g. the course of one or more biomarkers) will be illustrated by scatter/dotted plots and spaghetti plots, as appropriate. All statistical tests will be conducted using a two-sided significance level of α = 0.05. No adjustment for multiple testing will be performed. Given the exploratory nature of the study, all statistical test results will be interpreted as hypothesis-generating rather than confirmatory. Statistical analyses will be performed with R, The R Project for Statistical Computing with the latest version available (currently 4.5.3).

#### Interrater variability

Bland-Altman analysis will be performed on log-transformed volumes to obtain ratio-based limits of agreement. Reliability will be calculated using the intraclass correlation coefficient (ICC; two-way random effects model, absolute agreement, single measures) and Lin’s concordance correlation coefficient (CCC), both reported with 95% confidence intervals. ICC values will be interpreted according to established procedures [30]: <0.50 poor, 0.50–0.75 moderate, 0.75–0.90 good, and > 0.90 excellent.

## Data Availability

No datasets were generated or analysed during the current study.

## Abbreviations

Aβ: Beta-amyloid
ADC: Apparent Diffusion Coefficient
ADL: Activities of Daily Living
ASL: Arterial Spin Labeling
BSI: German Federal Office for Information Security
CCC: Concordance Correlation Coefficient
CSVD: Cerebral Small Vessel Disease
DECT: Digital Enhanced Cordless Telecommunications
DICOM: Digital Imaging and Communications in Medicine
DTI: Diffusion-Tensor Imaging
DWI: Diffusion-Weighted Imaging
EQ-5D: EuroQol 5 Dimension
eCRF: Electronic case report form
FA: Fractional anisotrophy
FLAIR: Fluid Attenuated Inversion Recovery
GCP: Good Clinical Practice
GDS: Geriatric Depression Scale
GFAP: Glial Fibrillary Acidic Protein
GSP: Good Scientific Practice
HbA1c: Glycated Hemoglobin
HDL: High-Density Lipoprotein
IADL: Instrumental Activities of Daily Living
ICC: Intraclass Correlation Coefficient
ICF: Informed Consent Form
ISCED: International Standard Classification of Education
ISCO: International Standard Classification of Occupations
KRITIS: Critical Infrastructures
LDL: Low- Density Lipoprotein
MARS: Multi-site Auto-segmentation and Registration of Subcortical White Matter Hyperintensities
MPRAGE: Magnetization-Prepared Rapid Gradient Echo
MoCA: Montreal Cognitive Assessment
MRI: Magnetic Resonance Imaging
mRS: Modified Rankin Scale
NfL: Neurofilament Light Chain
NIHSS: National Institutes of Health Stroke Scale
NPA: Neuropsychologic assessment
NVDU: Neurovascular Doppler ultrasound
PSMD: Peak width of skeletonized mean diffusivity
pTau217: Phosphorylated Tau 217
QoL: Quality of Life
RBANS: Repeatable Battery for the Assessment of Neuropsychological Status
STRIVE: STandards for ReportIng Vascular changes on nEuroimaging
SWI: Susceptibility-Weighted Imaging
TAP: Test of Attentional Performance
TOF-MRA: Time-of-Flight MR Angiography
VCAM-1: Vascular Cell Adhesion Molecule-1
VEGF: Vascular endothelial growth factor
WAI: Work Ability Index
WMH: White Matter Hyperintensities
